# Challenges in Coagulation Management in Neurosurgical Diseases: A Scoping Review, Development, and Implementation of Coagulation Management Strategies

**DOI:** 10.3390/jcm12206637

**Published:** 2023-10-20

**Authors:** Menno R. Germans, Jonas Rohr, Christoph Globas, Tilman Schubert, Alexander Kaserer, Giovanna Brandi, Jan-Dirk Studt, Matthias Greutmann, Katharina Geiling, Lotte Verweij, Luca Regli

**Affiliations:** 1 Department of Neurosurgery, University Hospital Zurich, Rämistrasse 100 (CAMPUS), 8091 Zurich, Switzerland; jonas.rohr@usz.ch (J.R.); luca.regli@usz.ch (L.R.); 2 Clinical Neuroscience Center, University Hospital Zurich, Rämistrasse 100 (CAMPUS), 8091 Zurich, Switzerland; christoph.globas@usz.ch (C.G.); tilman.schubert@usz.ch (T.S.); 3 Department of Neurology, University Hospital Zurich, Rämistrasse 100 (CAMPUS), 8091 Zurich, Switzerland; 4 Department of Neuroradiology, University Hospital Zurich, Rämistrasse 100 (CAMPUS), 8091 Zurich, Switzerland; 5 Institute of Anesthesiology, University Hospital Zurich, Rämistrasse 100 (CAMPUS), 8091 Zurich, Switzerland; alexander.kaserer@usz.ch; 6 Neurocritical Care Unit, Institute for Intensive Care Medicine, University Hospital Zurich, Rämistrasse 100 (CAMPUS), 8091 Zurich, Switzerland; giovanna.brandi@usz.ch; 7 Department of Medical Oncology and Hematology, University Hospital Zurich, Rämistrasse 100 (CAMPUS), 8091 Zurich, Switzerland; jan-dirk.studt@usz.ch; 8 University Heart Center, Department of Cardiology, University Hospital Zurich, Rämistrasse 100 (CAMPUS), 8091 Zurich, Switzerland; matthias.greutmann@usz.ch; 9 Department of Geriatrics, University Hospital Zurich, Rämistrasse 100 (CAMPUS), 8091 Zurich, Switzerland; katharina.geiling@usz.ch; 10 Institute for Implementation Science in Health Care, University of Zurich, Universitätstrasse 84, 8006 Zurich, Switzerland; lotte.verweij@uzh.ch; 11 Centre of Clinical Nursing Science, University Hospital Zurich, Universitätstrasse 84, 8006 Zurich, Switzerland

**Keywords:** neurosurgery, blood coagulation disorder, review, implementation science

## Abstract

Bleeding and thromboembolic (TE) complications in neurosurgical diseases have a detrimental impact on clinical outcomes. The aim of this study is to provide a scoping review of the available literature and address challenges and knowledge gaps in the management of coagulation disorders in neurosurgical diseases. Additionally, we introduce a novel research project that seeks to reduce coagulation disorder-associated complications in neurosurgical patients. The risk of bleeding after elective craniotomy is about 3%, and higher (14–33%) in other indications, such as trauma and intracranial hemorrhage. In spinal surgery, the incidence of postoperative clinically relevant bleeding is approximately 0.5–1.4%. The risk for TE complications in intracranial pathologies ranges from 3 to 20%, whereas in spinal surgery it is around 7%. These findings highlight a relevant problem in neurosurgical diseases and current guidelines do not adequately address individual circumstances. The multidisciplinary COagulation MAnagement in Neurosurgical Diseases (COMAND) project has been developed to tackle this challenge by devising an individualized coagulation management strategy for patients with neurosurgical diseases. Importantly, this project is designed to ensure that these management strategies can be readily implemented into healthcare practices of different types and with sustainable integration.

## 1. Introduction

Neurosurgical diseases and their surgical treatment carry a high risk for bleeding and thromboembolic (TE) complications, often leading to a deterioration in neurological status [1,2,3,4]. Due to the potentially devastating consequences of intracranial or intraspinal bleeding, these types of bleeding are assessed as “major bleeding in surgical patients” according to the International Society on Thrombosis and Haemostasis. Various types of bleeding complications occur within this population, such as progressive hemorrhagic injury in traumatic brain injury (TBI), rebleeding after aneurysmal subarachnoid hemorrhage (aSAH), intraoperative and postoperative hemorrhage after elective or emergency craniotomies, and postoperative epidural hematoma in spine surgery. Each complication carries its distinct risk profile and impact on patient outcomes. This risk profile is influenced by comorbidities, preoperative anticoagulation, or antiplatelet therapy, but also by the disease itself, which can induce coagulopathy [5,6,7].

In contrast to bleeding complications, TE complications such as stroke, myocardial infarction (MI), deep venous thrombosis (DVT) and pulmonary embolism (PE) also affect the overall outcome of neurosurgical patients, even beyond their hospital stay [8,9,10].

Balancing the prevention of TE complications with the minimization of bleeding risks in patients with neurosurgical diseases is a delicate and challenging task. Developing optimal strategies for coagulation management faces obstacles due to a lack of evidence-based protocols. This is likely because of the variety of neurosurgical diseases, the heterogeneity of comorbidities, and the use of preexisting medications. Currently, only a few guidelines on coagulation management in patients with neurosurgical diseases are available [11,12,13,14,15,16,17,18,19,20]. These guidelines have limitations as they only briefly address coagulation management for single neurosurgical diseases, focus on patients who do not require a neurosurgical intervention, and do not incorporate general risk assessment tools into the decision-making process.

The current aging population will cause an increased use of antithrombotic medications, including anticoagulation and antiplatelet therapy, andolder individuals are more susceptible to coagulation disorders due to various age-related factors [21]. Additionally, reliable risk assessment tools for the prediction of TE complications in different comorbidities are widely available and applied in clinical decision-making. However, these tools are rarely integrated into the coagulation management of neurosurgical diseases. The importance of a coagulation management strategy in neurosurgery has gained prominence in recent years with publications of disease-specific guidelines and the establishment of a taskforce for the start of antithrombotic medications following TBI [12,17,22]. Finally, one of the objectives of the World Health Organization’s strategy on the safety and availability of blood products, known as “Effective implementation of patient blood management to optimize clinical practice of transfusion” also advocates for an optimized strategy for the use of blood products in neurosurgical patients. In summary, the following reasons underscore the need for investigating coagulation management strategies in neurosurgical diseases:

- existing guidelines on coagulation management have several limitations;
- an aging population with increased use of antithrombotic medications;
- a higher incidence of coagulation-associated postoperative complications due to the aging population;
- the availability of prediction models for the development of thromboembolic complications that are seldom incorporated into decision-making processes;
- the potential profound impact of reducing bleeding and thromboembolic complications on the healthcare and socioeconomic systems, and;
- the global objective of the WHO is to implement patient blood management

The lack of evidence on this challenging topic highlights the importance of studies investigating optimal coagulation management for patients with various neurosurgical diseases.

This publication presents a scoping review of the literature and addresses the challenges and knowledge gaps in the management of coagulation disorders in neurosurgical diseases. Additionally, it introduces a research project aimed at systematically summarizing available evidence, generating new evidence related to optimal coagulation management in neurosurgical diseases, and developing recommendations on sustainable integration and implementation of this evidence into healthcare practice.

## 2. Scoping Review of Literature and Identification of Knowledge Gaps

### 2.1. Literature Search

**Figure 1:**
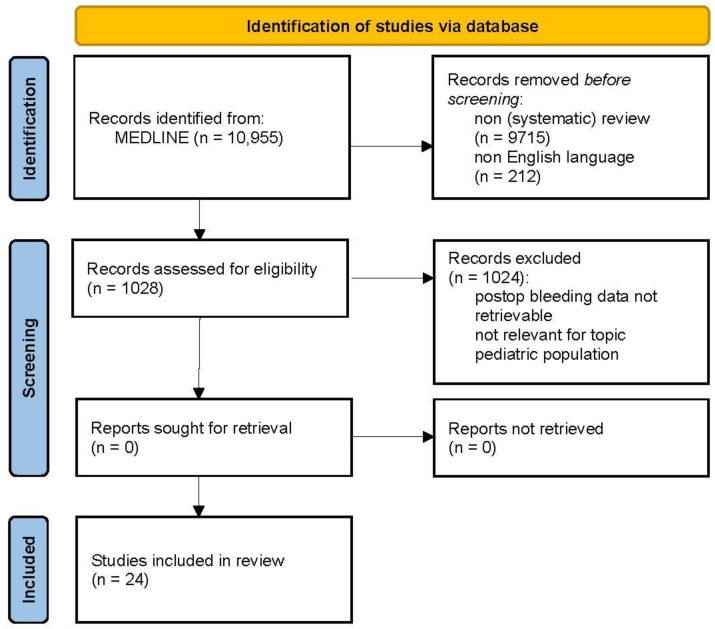
PRISMA flow diagram of included studies regarding bleeding complications in neurosurgery.

**Figure 2:**
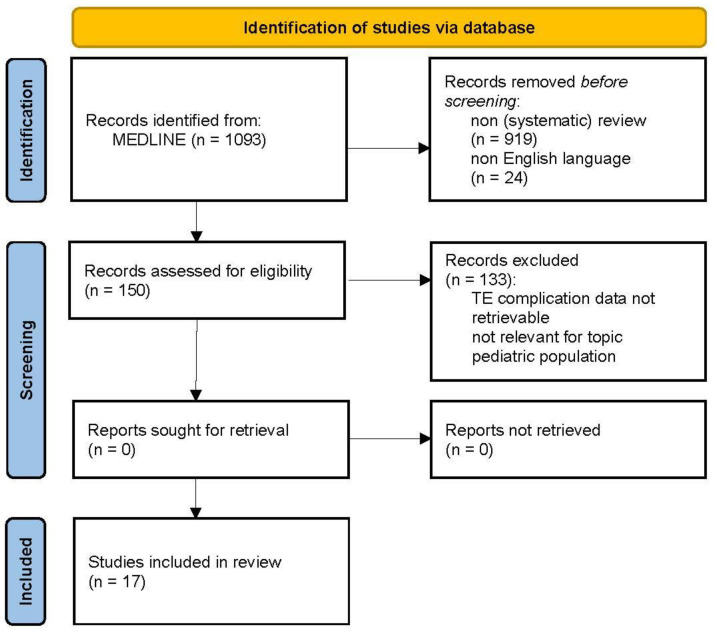
PRISMA flow diagram of included studies regarding thromboembolic (TE) complications in neurosurgery.

The protocol of this study was guided by the PRISMA-ScR statement [23]. The current literature was searched in MEDLINE (PubMed) using search terms relevant to the different topics: “(bleeding[MeSH Terms])”, “(thromboembolism[MeSH Terms]) OR (deep venous thrombosis[MeSH Terms])” combined with “neurosurgery[MeSH Terms])” from database inception until June 2023. Only (systematic) reviews on adults and articles written in English were assessed for eligibility. Search results on bleeding (n = 1028) and TE complications (n = 150) were screened by two authors (MRG, JR) for relevance based on title and abstract screening. Data on bleeding and TE complications in patients with various neurosurgical diseases were extracted and assessed for relevance to the topic of this review by the leading author. Finally, 24 and 17 articles were selected to summarize the literature on bleeding and TE complications, respectively. See Figure 1 and Figure 2 for a flow diagram of the selection processes. The type of complications are presented for different intracranial pathologies and spinal surgery separately with a summary of (range of) proportions.

### 2.2. Bleeding Complications

Complications arising from bleeding in neurosurgical patients are particularly concerning due to often devastating damage to the central nervous system [1,2,4]. These complications can manifest before treatment (e.g., rebleeding in aSAH), during surgery (e.g., excessive blood loss), and in the postoperative period (e.g., epidural or subdural hematoma). See Table 1 for an overview of all possible bleeding complications. While some bleeding complications result from coagulopathy induced by the disease itself, such as in TBI and aSAH, others are triggered by preoperative antithrombotic therapy, including anticoagulation and antiplatelet therapy [5,6,7].

Most bleeding complications prior to treatment are observed in TBI, due to the he-morrhagic expansion of a contusion or the progression of a subdural or epidural hematoma, and in aSAH with a sudden rebleeding of an intracranial aneurysm. These complications can lead to additional brain injury through direct destruction or compression of brain structures, brain hypoperfusion, or refractory high intracranial pressure. The incidence of progressive hemorrhagic injury in TBI varies between 20 and 60%, while the rebleeding rate in aSAH is about 10% per 24 h, even when following a protocol for emergency aneurysm treatment [24,25].

Intraoperative bleeding complications, such as diffuse hemorrhage and excessive blood loss, often result from coagulation disorders. These are usually caused by the brain pathology itself or by preoperative antithrombotic therapy. Therefore, assessing the coagulation system is imperative before starting the incision whenever possible. Several questions need to be addressed during the preoperative screening to identify disturbances in the coagulation system (see Table 2). Additionally, conventional laboratory tests can objectively evaluate the function of the coagulation system. In emergency situations where there is insufficient time for conventional laboratory testing, point-of-care tests offer a viable alternative [26]. If a disturbance of the blood coagulation at the time of surgery is detected, efforts should be made to investigate and optimize the cause(s) whenever possible, as intraoperative coagulation disorders are associated with postoperative complications and worse outcomes [1,2,4].

Even when clinically adequate hemostasis is achieved at the end of surgery, postoperative bleeding remains a concern. Given the central nervous system’s sensitivity to compression and hypoperfusion, combined with the fact that bleeding in the brain or spine usually occurs in a closed compartment with limited room for expansion, even small-volume hemorrhage can lead to severe temporary or permanent neurological deficits. The risk of postoperative bleeding following elective craniotomy is approximately 3%, whereas in other indications (e.g., trauma and intracranial hemorrhage) the risk is significantly higher, ranging from 14 to 33% [28,29,30,31]. Postoperative bleeding requiring emergency reoperation after spinal surgery occurs in roughly 0.5 to1.4% of cases and is strongly associated with permanent postoperative neurological deficits [32,33,34].

### 2.3. Thromboembolic Complications

The majority of thromboembolic (TE) complications associated with neurosurgical diseases encompass stroke, myocardial infarction (MI), pulmonary embolism (PE), or deep venous thrombosis (DVT). These complications may not only manifest during the primary hospitalization but can also occur after hospital discharge [10,35,36]. TE complications often lead to the readmission of patients to departments other than neurosurgery, which may result in the neurosurgeon not having an awareness of the patient’s postoperative course. Therefore, when studying TE complications, it is crucial to investigate the complications in the months following the neurosurgical treatment without limiting the analysis to the initial admission alone. Furthermore, the type of neurosurgical disease strongly affects the risk of developing TE complications. This can be attributed to differences in exposure to prolonged immobilization, vascular endothelial damage, postoperative inflammatory status after major surgery, and the presence of cancer. For an overview of TE complications in different neurosurgical diseases, see Table 3.

It is well-established that the risk for TE complications is associated with several risk factors. Prediction models for various types of TE complications are available and can be applied in clinical practice. Some widely accepted models include the ABCD2 and CHA2DS2-VASc-score in stroke [45,46], as well as the Caprini-score and Khorana model in PE and DVT [47,48]. For assessing the risk of TE complications and managing antiplatelet and anticoagulation therapy in patients with pre-existing cardiac conditions, risk assessment and management strategies, along with algorithms, have been proposed [49]. While these scores and models provide a general indication of TE risk, their association with various neurosurgical diseases remains largely unknown. Moreover, TE complications pose an elevated risk to older individuals, due to various age-related factors, including age-related inflammation, reduced venous flow, hypercoagulability, endothelial dysfunction, polypharmacy, comorbidities, and environmental factors [50]. These factors are only partially, if at all, incorporated into existing prediction models.

### 2.4. Available Guidelines

There are only a limited number of guidelines available for coagulation disorders in neurosurgical diseases. Most of these focus on intracranial hemorrhages, including traumatic or spontaneous intracerebral hemorrhage, and subarachnoid hemorrhage [12,14,15,16,17,19,20]. The recommendations, however, are intended in most cases for patients who do not require a neurosurgical intervention, like external ventricular drainage or craniotomy. Consequently, these guidelines are less relevant for the majority of the neurosurgical population.

A critical appraisal of guidelines for the use of antithrombotic therapy in spine surgery highlighted the uncertainty regarding optimal practices for balancing the risk of thromboembolism against that of bleeding [18].

There is a greater body of evidence for the prevention of TE complications in neurosurgical patients available, which is summarized in general or disease-specific guidelines [11,13,20]. Nevertheless, these guidelines do not incorporate current generally accepted models for the prediction of TE complications, making them less suitable for application in individual circumstances.

### 2.5. Knowledge Gaps

This scoping review of the literature demonstrates the prevalence and significant concern surrounding bleeding and TE complications in the neurosurgical population. Given the variety of neurosurgical diseases, different comorbidities, and an aging patient population with a concurrent increase in the use of antithrombotic medications, establishing guidelines that can be applied to the neurosurgical population is challenging. A more individualized approach in coagulation management, considering the numerous factors that influence not only treatment-associated but also long-term complications, may be more appropriate for patients with neurosurgical diseases. These factors explain the substantial knowledge gap in the literature for optimal coagulation management of neurosurgical patients.

Furthermore, translating knowledge into healthcare practice is a complex and time-consuming process, yet it remains the most effective means of improving patient outcomes. Research has indicated that integrating evidence into healthcare practice can take up to 17 years [51]. The investigation of the barriers and facilitators of the implementation of available evidence in neurosurgical diseases has hardly been investigated before, representing a significant gap in the literature that requires further exploration and clarification.

Assuming that the current literature contains medium to high-quality evidence supporting an individualized approach to patients with various neurosurgical diseases, and recognizing the importance of the implementation of knowledge into healthcare practice, we have initiated a research project to address this clinical problem.

### 2.6. The COMAND Project

With the goal of developing an individualized coagulation management strategy for patients with various types of neurosurgical diseases, we have established a research project, called COagulation MAnagement in Neurosurgical Diseases (COMAND). This project involves a multidisciplinary team of medical experts of different disciplines (neurosurgery, neurology, neuroradiology, neuro-intensive care, anesthesiology, hematology, cardiology and geriatrics) at the University Hospital Zurich, Switzerland. Additionally, it includes epidemiologists, an implementation scientist and representatives from patient advisory boards. The scope of the project is defined as follows: “In order to reduce coagulation disorder-associated complications, we aim at sustainable integration of moderate to high-quality evidence for the management of coagulation disorders and thromboembolic complications in neurosurgical patients within the next five years”.

The COMAND project has been initiated with the purpose of compiling a comprehensive overview of the current evidence regarding the optimal management of coagulation disorders in the neurosurgical population. The quality of this evidence will be assessed using the Grading of Recommendations Assessment, Development and Evaluation (GRADE) approach [52]. The aim is to make this information accessible to the global community through an open-access platform. Scientific gaps will be identified and subsequent studies to investigate clinically relevant questions will be designed and launched. To ensure the success of these studies within a reasonable timeframe, active national and international cooperation is pursued.

Ultimately, the research group wants to achieve a lasting transformation in healthcare practice where necessary and good adherence to management protocols by physicians across various hospitals and healthcare settings. To achieve this, the project not only focuses on coagulation management strategies and the creation of evidence but also on addressing the conditions required to realize change in healthcare practice, along with the necessary resources, to have a significant impact on real-world implementation [53].

In short, the key objectives of the COMAND project are:

1. summarize existing evidence on the optimal management of coagulation disorders and prevention of thromboembolic complications for different neurosurgical diseases, and identify knowledge gaps;
2. generate new evidence on the optimal management of coagulation disorders and the prevention of thromboembolic complications to cover identified knowledge gaps, and;
3. develop recommendations on the sustainable integration and implementation of the evidence-based guideline.

The investigated neurosurgical diseases are divided into four groups: intracranial tumor surgery; traumatic brain injury; intracranial hemorrhage, including intracerebral hemorrhage, subarachnoid hemorrhage and chronic subdural hematoma and spine surgery, divided into degenerative diseases, emergency procedures and intradural surgery. Each disease topic will be investigated for optimal management protocols both before and after neurosurgical treatment, irrespective of invasive treatment or not.

#### COMAND Project and Medical Research Council Framework

The COMAND project is considered a complex intervention in healthcare practice, based on a large number of relevant components involved in decision-making, different behaviors among physicians, different hospital policies, and the need for specific knowledge and skills [53]. Furthermore, the project involves both implementing existing evidence and generating new evidence, each of which demands distinct approaches to achieve sustainable integration in healthcare practice. To achieve these goals across various neurosurgical diseases, we decided to use the recently updated Medical Research Counsil (MRC) framework as a supportive framework [53]. The goal of the framework is not only to investigate the effectiveness of a specific treatment but also to assess whether it is acceptable, implementable, cost-effective, scalable and transferable across contexts [53]. The MRC framework serves as the umbrella framework for the entire COMAND project. Additional guiding frameworks will be specified within each individual study generated within this research project. For instance, an implementation science framework such as the updated Consolidated Framework for Implementation Research (CFIR) or the Tailored Implementation of Chronic Diseases (TICD) checklist may be relevant to guide the implementation study within this research project [54,55].

The COMAND project will focus on the following perspectives for each disease group:

Efficacy: Is there any moderate or high-quality evidence, assessed through the GRADE approach, that a specific coagulation management protocol yields better patient outcomes?

Effectiveness: Has a specific management protocol been applied in a real-world setting and to what extent did it achieve the intended effect?

Theory based: How did the specific management protocol lead to the intended effect, considering the interactions between bleeding complications and TE complications, the mechanisms of the management protocol, the contextual features that may influence those mechanisms, and how those mechanisms might influence the context?

Systems: How do the healthcare practice and management protocols adapt to each other?

The research group, along with relevant stakeholders, will identify contextual determinants that could be relevant to the implementation process of the coagulation management strategies. This implementation process will be tailored for each neurosurgical disease group, allowing for the development of strategies that facilitate optimal integration and implementation in healthcare practice.

The structure of the MRC framework is used to divide the project into four phases: (1) development or identification of the intervention, (2) feasibility and efficacy testing, (3) evaluation of effectiveness and (4) evaluation of implementation processes and outcomes. For a detailed explanation of the four phases, we refer to the article of Skivington et al. [53] The phases for the current project are explained in more detail below.

Identification of the complex intervention: changing the management of coagulation disorders in the neurosurgical population in various settings is complex. Developing a program theory (see above) and identifying key uncertainties are essential to evaluate the project effectively.

Feasibility: In this phase, necessary criteria are developed to assess the successful application of management protocols. If the program theory suggests that contextual or implementation factors might influence the acceptability, effectiveness, or cost-effectiveness of the management options, these questions should be considered. It will be debated, whether an evaluability assessment will be performed to define data that are relevant to assess process and outcome, and the options for designing the evaluation.

Evaluation: According to the updated MRC framework, the evaluation phase is defined as “going beyond asking whether an intervention works (in the sense of achieving its intended outcome), to a broader range of questions including identifying what other impact it has, theorizing how it works, taking account of how it interacts with the context in which it is implemented, how it contributes to system change, and how the evidence can be used to support decision making in the real world” [53]. Therefore, the outcome measures for the implementation of the COMAND project should be thoughtfully chosen to effectively assess project feasibility. These measures should primarily evaluate the system level (i.e., the hospital) rather than focusing on individuals or groups.

Implementation: During this phase, the implementation questions are developed, encompassing implementation strategies and outcomes such as service reach or acceptance, as well as contextual determinants that affect the project’s successful implementation.

## 3. Discussion

Bleeding and TE complications in the neurosurgical population are not only associated with risks for permanent neurological deficits but also for disruptions in other organ systems and readmission to acute healthcare systems. This scoping review summarizes current literature on the risk of bleeding and TE complications, revealing significant heterogeneity among different neurosurgical diseases. The variety of neurosurgical diseases and associated comorbidities complicates the implementation of general guidelines on coagulation management in neurosurgical diseases. As such, a more individualized approach to coagulation management with the focus on implementing tailored strategies appears the direction for future projects.

Patients with neurological deficits place a substantial burden on the healthcare system. Additionally, as a considerable proportion of the neurosurgical population consists of individuals in their productive working years, their impact on the socio-economic system is substantial. Improving the management of coagulation disorders in this population would not only have a profound impact at the individual level but also on the healthcare and socio-economic systems.

The vast availability of scientific evidence and improved collaboration among researchers opens possibilities for an individualized approach to specific and rare diseases. Translating the evidence into clinical practice, however, is estimated to take about 17 years [51]. In addition to generating high-quality evidence, equal attention should be given to the implementation of evidence in healthcare practice. It is worth noting that there has been limited exploration of the implementation of evidence in the field of neurological sciences. For instance, a review focusing on overall barriers and enablers in stroke care identified only ten relevant articles, and very recently, the first implementation study for a goal-directed care bundle after acute intracerebral hemorrhage was published [56,57]. This underscores the large research area to be discovered, especially within the neurosurgical community.

The MRC framework for developing and implementing complex interventions provides valuable guidance for achieving a sustainable integration of management strategies into healthcare practice. Without the attention to implementing evidence across various settings and among various physicians worldwide, the available moderate to high-quality evidence on coagulation management would probably not—or in a delayed fashion—reach physicians, potentially impeding improvements in healthcare practices.

This article does have certain limitations in terms of data collection and analysis. A structured systematic review of the literature and data extraction was not conducted according to a prespecified protocol. The main goal was to give a scoping overview of the current potentially relevant literature, and therefore, a systematic review was not within the scope of this work. However, it is important to note that the COMAND project has been designed to deliver a summary of data across various neurosurgical diseases. As such, systematic reviews on coagulation disorders and TE complications in neurosurgical diseases are planned for the near future. Other potential limitations of the COMAND project include the assumptions that there is some moderate to high-grade evidence in the literature and that the neurosurgical community supports new studies investigating coagulation management in neurosurgical patients. To address the latter concern, the COMAND project has been designed according to the MRC framework, which allows for the investigation and potential development of strategies for expedited and sustainable integration of evidence in healthcare practice. Applying the MRC framework to a project that combines existing evidence with the creation of new evidence is undoubtedly challenging. However, the successful execution of this COMAND project can only be achieved through productive collaboration among medical specialists, implementation scientists, epidemiologists and patients.

## 4. Conclusions

Bleeding and thromboembolic complications are common in the neurosurgical population and their risk depends on the type of disease and numerous influencing factors. It is, therefore, difficult to develop overarching guidelines for coagulation management in neurosurgical diseases. A more individualized approach may be more suitable for patients with neurosurgical diseases. The COagulation MAnagement in Neurosurgical Diseases (COMAND) project meets the individualized management in patients with various neurosurgical diseases. This project has been designed in a manner that allows for the implementation of these management strategies in healthcare practices of different types and with sustainable integration.

## Figures and Tables

**Table 1:** Overview of potential bleeding complications in various neurosurgical diseases.

	Bleeding Complication		
Disease Type	Before Treatment	During Surgery	After Treatment
Intracranial tumor surgery	intratumoral hemorrhage with extension to: epidural subdural intraparenchymal intraventricular	excessive blood loss (remote) intraparenchymal hemorrhage	intraventricular hemorrhage intracavitary hemorrhage intraparenchymal hemorrhage subdural hemorrhage epidural hemorrhage subcutaneous hemorrhage
Traumatic brain injury	intraventricular hemorrhage intraparenchymal hemorrhage subarachnoid hemorrhage subdural hemorrhage epidural hemorrhage subcutaneous hemorrhage		
Intracranial hemorrhage	intraventricular hemorrhage intraparenchymal hemorrhage subarachnoid hemorrhage (aneurysm rebleeding) subdural hemorrhage epidural hemorrhage		
Spine surgery	intramedullary hemorrhage intradural, extramedullary hemorrhage epidural hemorrhage	excessive blood loss injury of paraspinal vasculature	subdural hemorrhage epidural hemorrhage subcutaneous hemorrhage

**Table 2:** Questions that can be asked for assessment of a coagulation disorder (based on HEMSTOP questionnaire [27]).

1. Have any medications affecting clotting been used in the last 14 days?
2. Have you ever consulted a doctor or received treatment for prolonged or unusual bleeding (such as nosebleeds, minor wounds)?
3. Do you experience bruises/hematomas larger than 2 cm without trauma or severe bruising after minor trauma?
4. After a tooth extraction, have you ever experienced prolonged bleeding requiring medical/dental consultation?
5. Have you experienced excessive bleeding during or after surgery?
6. Is there anyone in your family who suffers from a coagulation disease (such as hemophilia, von Willebrand disease, etc.)?
For females:
1. Have you ever consulted a doctor or received a treatment for heavy or prolonged menstrual periods (contraceptive pill, iron, etc.)?
2. Did you experience prolonged or excessive bleeding after delivery?

**Table 3:** Overview of thromboembolic complications in various neurosurgical diseases.

	Type of TE Complication	Risk (%)	Interval
Craniotomy [37,38]	VTE	3.2–7.8	n.a.
	MI	2.5	n.a.
	Stroke	3.6	n.a.
Intracranial tumor surgery [39,40]	VTE	3.0–17.0	<30 days ^1^
Malignant brain tumor [10]	VTE	3.5	<30 days
Intracranial meningioma [35]	VTE	7.2	<60 days
TBI [41]	VTE	3.8	<60 days
SAH [41,42]	VTE	6.7–21	<60 days ^2^
Intracerebral hemorrhage [41]	VTE	2.9	<60 days
Spinal surgery [34,43]	VTE	5.1	n.a.
	MI	1.3	n.a.
	Stroke	0.9	n.a.
Surgery in general [36,44]	IE	4.4	<30 days
	VTE	3.3	<84 days

VTE: venous thromboembolism (i.e., deep venous thrombosis and pulmonary embolism), IE ischemic events (VTE, MI and stroke), MI myocardial infarction, n.a. not applicable, SAH subarachnoid hemorrhage, TBI traumatic brain injury, TE thromboembolic. ^1^ n.a. for reference Smith [40]. ^2^ <30 days for reference Liang [42].

## Data Availability

Will individual participant data be available (including data dictionaries)? Yes. What data in particular will be shared? Data openly available in a public repository that issues datasets with DOIs. What other documents will be available? Introduction and study protocols. When will data be available (start and end dates)? Immediately following publication; no end date. With whom? Researchers who provide a methodologically sound proposal. For what types of analyses? To achieve aims in the approved proposal.
